# An Evaluation of the Role of Sensory Drive in the Evolution of Lake Malawi Cichlid Fishes

**DOI:** 10.1155/2012/647420

**Published:** 2012-06-21

**Authors:** Adam R. Smith, Moira J. van Staaden, Karen L. Carleton

**Affiliations:** ^1^Department of Biology, Indiana University, 1001 East 3rd Street, Jordan Hall 142, Bloomington, IN 47405, USA; ^2^Department of Biological Sciences, Bowling Green State University, 217 Life Sciences Building, Bowling Green, OH 43403, USA; ^3^Department of Biology, University of Maryland, 1210 Biology-Psychology Building, College Park, MD 20742, USA

## Abstract

Although the cichlids of Lake Malawi are an important model system for the study of sensory evolution and sexual selection, the evolutionary processes linking these two phenomena remain unclear. Prior works have proposed that evolutionary divergence is driven by sensory drive, particularly as it applies to the visual system. While evidence suggests that sensory drive has played a role in the speciation of Lake Victoria cichlids, the findings from several lines of research on cichlids of Lake Malawi are not consistent with the primary tenets of this hypothesis. More specifically, three observations make the sensory drive model implausible in Malawi: (i) a lack of environmental constraint due to a broad and intense ambient light spectrum in species rich littoral habitats, (ii) pronounced variation in receiver sensory characteristics, and (iii) pronounced variability in male courtship signal characteristics. In the following work, we synthesize the results from recent studies to draw attention to the importance of sensory variation in cichlid evolution and speciation, and we suggest possible avenues of future research.

## 1. Introduction

The cichlid faunas of the east African rift lakes encompass some of the most species-rich extant vertebrate radiations [1]. Amongst the flocks from Lakes Malawi, Tanganyika, and Victoria, the radiation in Lake Malawi is the largest and most diverse in terms of species number [2]. Given the young age of the radiation (~1-2 mya, [3, 4]), Malawi cichlids are a valuable system for the study of both rapid speciation and niche evolution [5]. Albertson et al. [6] first laid out a model for species radiation in three stages: (i) habitat diversification, (ii) trophic diversification, and (iii) sexual selection. Sexual selection is likely a primary mechanism for speciation within genera, as is evidenced by clear differences in male breeding color displayed by ecologically similar congeners. Many studies have demonstrated that females can distinguish between conspecific and congeneric males based on nuptial coloration alone [7, 8], although cues from other sensory modalities are likely in play as well [9–11].

Despite the apparent importance of sexual selection in the radiation of Malawi cichlids, the evolutionary processes underlying diversification in female sensory sensitivities and mate preferences are as yet unknown. Given current knowledge regarding cichlid sensory systems [12–14], courtship signal structure [9, 15–17], and female behaviors [7, 18, 19], the experimental data is available to critically evaluate models of signal diversification and mate choice. In the following paper, we will synthesize contemporary cichlid research, particularly as it applies to cichlid visual systems in Lake Malawi. Furthermore, we will discuss some potential mechanisms underlying sexual selection in Lake Malawi.

## 2. Sensory Drive and the Stages of Communication

A linkage between sensory evolution and male courtship signals was eloquently laid out by Ryan and Rand [20] in their sensory exploitation model. This model was expanded upon by Endler [21], who incorporated environmental influences to develop an evolutionary model known as the sensory drive hypothesis. The sensory drive model of evolution focuses on selection for the three primary steps of communication, which can be broadly defined as (i) passive or active emission of a signal by a signaler, (ii) transmission of the signal through the environmental channel, and (iii) perception of the signal by the receiver. 

Sensory drive orders these three primary communication steps in a hierarchy to define a cascade of selection processes that link the environment, receiver sensory capabilities, and the properties of communicative signals. As a result, (i) the environmental transmission channel modulates signal intensity and fidelity, (ii) the sensory capabilities of the receiver should evolve such that greatest sensitivity is achieved at the region of highest environmental transmission, and (iii) signal properties should then evolve to match this sensory system (see Figure 1 in [21]). The model encompasses selective forces imposed by diverse ecological factors such as food detection, predation, and microhabitat choice for male displays. It is important to recognize that sensory drive emphasizes the role of the environmental transmission channel as a selective force constraining sensory and signal evolution [22].

## 3. Cichlids, Sensory Systems and Models of Sexual Selection

When considering sexual selection in Lake Malawi cichlids, it is useful to compare it with sexual selection in the sister flock in Lake Victoria. Much like Malawi, Lake Victoria harbors a young (<500,000 years; [3]) cichlid radiation. Anthropogenic eutrophication (primarily due to agricultural runoff) threatens species diversity in Victoria due to the breakdown of species-recognition barriers in murky waters [23]. This effect highlights the environmental constraint on sensory capacities and signals and the importance of visual communication in speciation processes. The dim, narrow-spectrum light environment in Lake Victoria strongly constrains both visual properties and male nuptial displays. It can also limit the depth range of haplochromines, increasing interspecific space use along the depth gradient [24]. Reduced visibility can lead to a breakdown of species recognition, leading to a loss of species diversity through hybridization (although it may promote speciation in certain instances; see [25]). This eutrophication process and the hydrology of Victoria as a whole have also imposed extreme selection on cichlid visual systems and mate selection mechanisms [26]. As a result, the cichlids of Lake Victoria are an important model system for sensory drive based on behavioral and LWS opsin sequence data [27]. 

Pronounced differences in the visual environments of Lakes Victoria and Malawi suggest that it is not necessarily appropriate to extrapolate findings from one lake to the other. Unlike Victoria, Malawi is a clear-water lake with high-intensity light in shallow waters and a broad transmission spectrum. While the global light spectrum found in Lake Malawi does shape the overall visual sensitivities used by cichlids in those lakes, the light spectrum typically changes gradually between habitats or with depth. Consequently, it is unlikely that the environmental transmission channel in the shallow littoral environments of Lake Malawi exerts quite the same constraining selective pressure on sensory systems and signals that it does in Lake Victoria.

## 4. Does the Ambient Light Environment in Lake Malawi Constrain Visual Communication?

**Figure 1:**
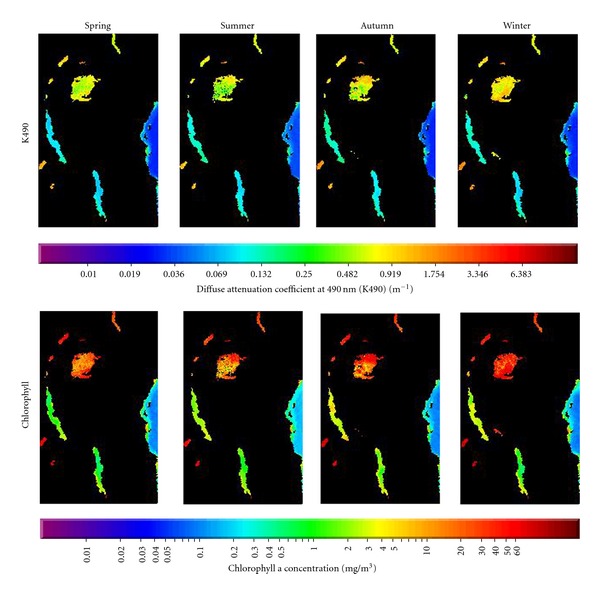
Seasonal satellite images for the three major African rift lakes, with Victoria at the top, Tanganyika in the middle, and Malawi at the bottom. The higher attenuation coefficients (K490, top row) and chlorophyll concentration (bottom row) estimates demonstrate that Lake Victoria is more turbid than the other lakes and, therefore, has a different fundamental ambient light environment. Lakes Tanganyika and Malawi are fairly similar and have much clearer waters than Victoria. Images constructed from averaged SEAWIFS data for the years 1998–2002. The large body of water on the right is a portion of the Indian Ocean that borders the African horn.

The initial (and causative) step in the sensory drive model emphasizes the role of the environmental transmission channel in shaping sensory sensitivities. This raises the question, how often do environmental transmission channels impose significant selection on sensory systems? Satellite imagery of light transmission through the waters of Lakes Malawi and Victoria illustrates the fundamental differences in the visual environments of these two lakes (Figure 1). The turbid waters of Lake Victoria optimally transmit longer wavelengths (orange and red), while the typically clear waters of Lake Malawi optimally transmit intermediate wavelengths (blue and green). Although both habitats are subject to short-term seasonal perturbations, the fundamental difference in environmental transmission is stable throughout the year (Figure 1). Therefore, Malawi offers us the opportunity to investigate signal constraint in a light environment substantially different to that studied in Victoria.

**Figure 2:**
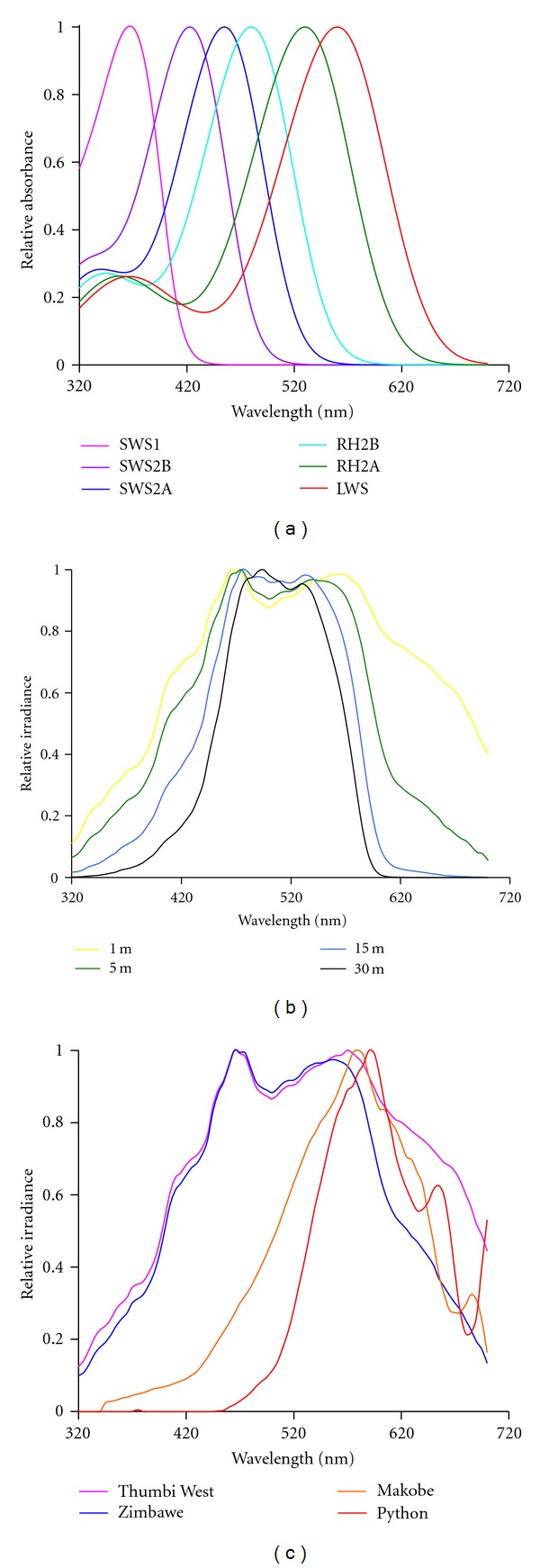
Comparison of cichlid cone opsin absorbance spectra in various environmental light environments. (a) Absorbance spectra for the six primary classes of cichlid cone opsins. (b) Relative irradiance measures of downwelling light at various depths at the Zimbawe Rock in Lake Malawi. (c) Comparison of the relative irradiance spectra for two Lake Malawi habitats (Thumbi West and Zimbawe) and two Lake Victoria habitats (Makobe and Python). Relative irradiance represents the proportion of total downwelling irradiance (photons/cm/s^2^) normalized to the wavelength of maximum transmission.

The tendency of blue-green wavelengths to transmit well in Lake Malawi is significant because these wavelengths correlate well with the absorbance of several cichlid visual pigments. The cichlid fishes of Lake Malawi possess seven distinct cone opsin genes, which we group into six functional categories (SWS1—ultraviolet, SWS2B—violet, SWS2A—blue, RH2B—blue—green, RH2A*α* and *β*—green, LWS—red; Figure 2(a)). Because of the spectral distribution of these pigment types, the blue-green wavelengths that are optimally transmitted in Lake Malawi closely match the area of predicted peak sensitivity for the Malawi cichlid visual system, particularly for the medium- and long-wavelength sensitive double cones (which express the RH2B, RH2A, and LWS opsin genes). This match is quite consistent and extends through the known depth distribution of many species ([14]; Figure 2(b)). However, the red-shifted light environments in Lake Victoria tend towards the long-wavelength end of the cichlid visual pigments (Figure 2(c)). 

**Figure 3:**
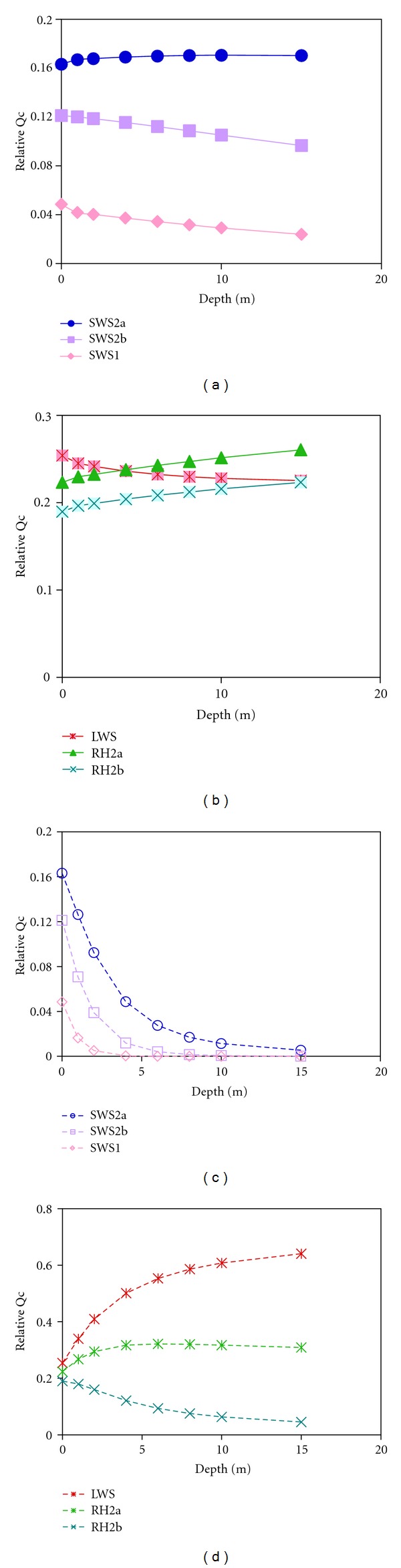
The relative quantum catch various opsin pigments for sample environments in Lakes Malawi and Victoria along depth gradients. The relative quantum catch of both short- (a) and long-wavelength (b) pigments is relatively constant in Lake Malawi. In Lake Victoria the short-wavelength pigments rapidly lose the ability to catch light with depth (c) while the relative quantum catch of the LWS pigment increases rapidly (d). Relative Qc represents the quantum catch (photons) of each cone pigment normalized to the total quantum catch for all pigments.

**Figure 4:**
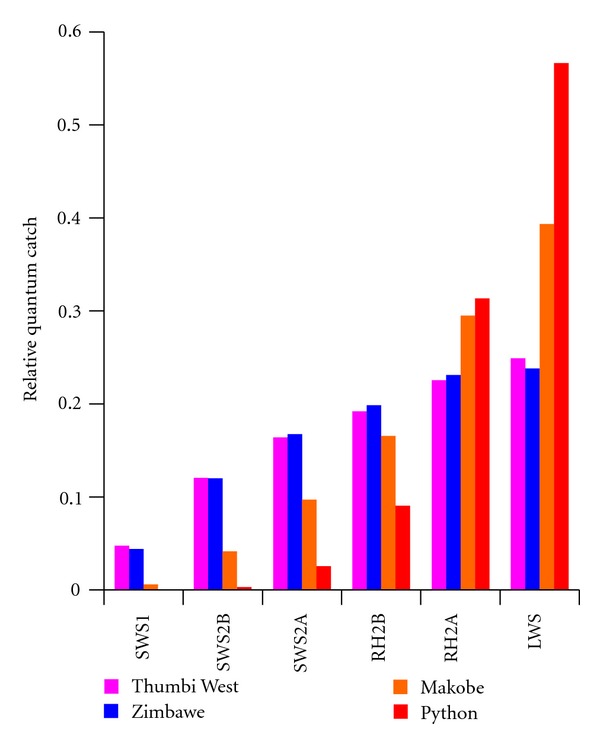
Absolute (a) and relative (b) cone opsin quantum catch comparisons distinct geographical locations in Lake Malawi (Thumbi West and Zimbawe) and Lake Victoria (Makobe and Python). Relative quantum catch represents the quantum catch (photons) of each cone pigment normalized to the total quantum catch for all pigments.

Smith et al. [14] used models of luminance sensitivity to predict differences in the total quantum catch of various visual systems in a given environment. These models highlighted the minimal impact of the spectral environment on cichlid vision in Lake Malawi, over a range of depths.  Figure 3 shows how quantum catch of the six classes of cichlid visual pigments varies with depth in both Lakes Malawi and Victoria [12]. These are calculated using

(1)Qi=∫I(λ)Tw(λ,d)R(λ)dλ,

where *I*(*λ*) is the solar light spectrum at the water surface as a function of wavelength *λ*,  *T*_w_(*λ*, *d*) is the spectral transmission properties of the water at depth *d*, and *R*(*λ*) is the absorption spectrum of the visual pigments calculated based on Govardovskii et al. 2000 [28]. We calculated these at depths up to 15 m, typical for the range where we collect fish. We then normalized the quantum catch calculations to reveal the relative tradeoffs between the different pigments with depth. In Malawi, the largest change occurs in the SWS1 pigment, with a decrease in quantum catchvaluesby a factor of 2 (Figure 3). The quantum catch of other visual pigments only varies by 5–15% over this 15 m depth range suggesting their quantum catch are all relatively good across this depth range. By contrast in Lake Victoria, the quantum catch for SWS1, SWS2B, and SWSA at 15 m decrease by 10^−6^, 10^−3^, and 0.03 relative to that at the surface. The relative quantum catch of LWS actually increases by 2.5 times over this depth range, supporting the idea that LWS is key to visual sensitivity in Lake Victoria. This highlights the fundamental differences in the light transmission properties of the two lakes as it applies to stimulating the cichlid cone visual pigments (Figure 4). It is also worth noting that absolute visual catch in Malawi can be 100 x greater than that in Victoria for similar depths due to greater availability of light (Figure 3), so that dim lighting is unlikely to have the same effects in Lake Malawi that it has in Lake Victoria.

In essence, the ambient light environments in Victoria and Malawi exhibit diametrically opposite effects on the cichlid visual system. In Lake Victoria, the environmental transmission spectrum skews towards longer wavelengths, creating selective pressure favoring increased long-wavelength sensitivity with depth as we have previously observed [12, 29]. However, in Lake Malawi, the environment tends towards the center of the visual spectrum, which is an area where several of the cichlid visual pigments exhibit high quantum catch values. The relatively constant quantum catch of visual systems with differential opsin expression patterns suggests that changes in gene expression patterns do not confer improved luminance detection in littoral habitats [14]. Therefore, the broad signal transmission channel in Malawi does not likely impose significant selection pressure on the chromatic visual system that would select for or against changes in opsin expression patterns. Because we observe all possible opsin expression patterns in a variety of species cooccurring at the same shallow depth in the same habitat [12], we cannot explain this diversity simply from the light transmission properties in the clear waters of Lake Malawi. This result is inconsistent with the first tenet of sensory drive.

## 5. Receiver Systems: Variability in the Visual System

The second tenet of the sensory drive hypothesis centers on the characteristics of the sensory system of the receiver. More specifically, it predicts that the diversification in sensory systems results in different degrees of signal discrimination. Over time, this can lead to divergent responses to signals and subsequent speciation. To some extent, this appears to be true in Lake Malawi. Different species in the lake generally express subsets of the six opsin classes (grouping RH2A*α* and *β* as RH2A), and these combinations have been termed “templates” [30]. These templates typically involve expression of three of the genes and occur as three primary types: (i) short—(SWS1, RH2B, RH2A), (ii) medium—(SWS2B, RH2B, RH2A), and (iii) long—(SWS2A, RH2A, LWS) sensitive gene sets [30]. 

However, significant variation within these primary templates has been found [14, 30]. This variability can manifest either as a quantitative variation in the relative expression of different opsin genes or as a qualitative shift from trichromatic to tetrachromatic opsin expression where additional genes are expressed [14]. This intraspecific variation has been found in the wild, both along depth gradients (5 m versus 20 m) and across geographically distinct locales, and appears to be largely independent of the ambient light environment. As such, species-specific expression profiles appear to be far from being stable or predictable in a quantitative sense.

**Figure 5:**
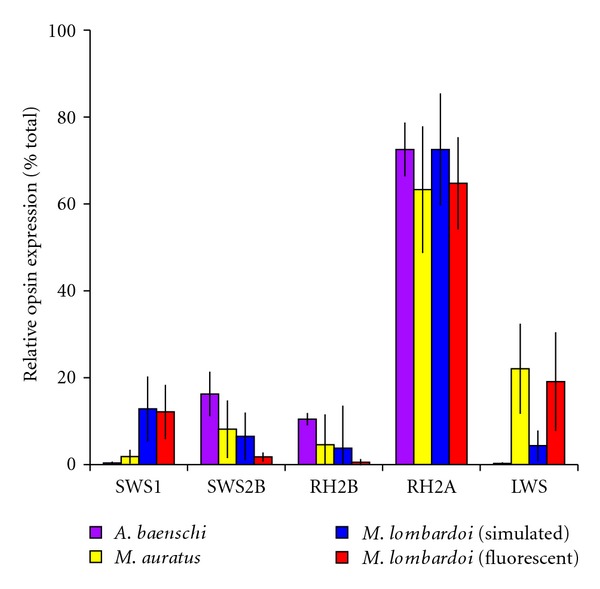
Gene expression patterns for fishes raised under laboratory conditions. *Aulonocara baenschi* and *Melanochromis auratus* both represent a primary medium-wavelength sensitive palette (SWS2B, RH2B, RH2A) in the wild. *A. baenschi* retains this template in the lab, while *M. auratus* develops substantial quantitative variation in LWS expression. *Metriaclima lombardoi* represents a short-wavelength sensitive palette (SWS1, RH2B, RH2A) in the wild but displays substantial variation in the expression of both the SWS2B and LWS genes in the laboratory. Furthermore, expression patterns for *M. lombardoi* in the lab are influenced by whether the fish are raised under fluorescent lighting or simulated sunlight. The SWS2A gene was omitted because it is not expressed in any of the groups depicted. Relative opsin expression represents the gene expression measure for each cone opsin gene normalized to the total measured opsin expression. Error bars indicate group standard deviations.

**Figure 6:**
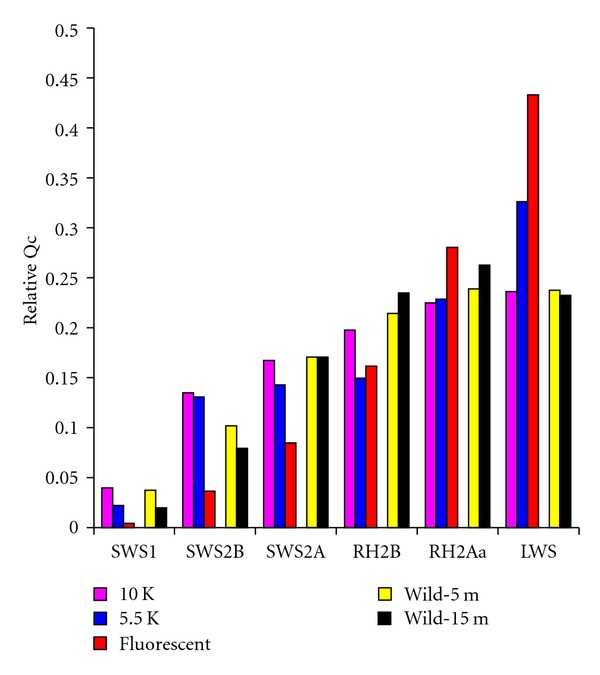
Relative quantum catch calculations for each of the primary cone opsin pigment classes for different light environments. The 10 K and 5.5 K bulbs were combined for a single simulated sunlight treatment, and pilot trials indicated no difference between the two in their effects on gene expression. However, the combination of these two had significant impact on gene expression when compared to fish raised under fluorescent lighting. Furthermore, the absolute difference in relative catch across treatments in the lab required to induce plasticity is much greater than that observed along the wild depth gradients where Smith et al. [14] collected samples for studies on wild expression plasticity. Relative Qc represents the quantum catch (photons) of each cone pigment normalized to the total quantum catch for all pigments.

While much of the large-scale variation in opsin expression between the cichlid templates has been shown to be genetic [31], there is some evidence for subtler shifts in opsin expression due to environmentally induced plasticity. Inducible opsin expression plasticity was first observed by Hofmann et al. [30] when F1 progeny reared in the laboratory were found to have expression profiles that differed from their wild-caught parents, with the effect being most apparent in the expression of the SWS2B and LWS genes. The plasticity is manifested as quantitative variation in genes that would otherwise be expressed at low levels, rather than changes to the trichromatic expression template (illustrated in Figure 5). Hofmann et al. attributed the plasticity to the differences between the natural and laboratory lighting environments, but no attempts to rescue wild-type expression patterns in the lab were performed. Prior work in bluefin killifish (*Lucania goodei*) has demonstrated that variations in the ambient light environment can induce a plastic response in opsin gene expression [32], and the laboratory-manipulated spectra that induce plastic responses can be tied to specific (clear or tannin-stained) natural environments. A similar experiment performed by Smith et al. [33] with Malawi cichlids found that by manipulating the ambient light environment opsin expression plasticity could be induced through development in some species but not others. In plastic species, fish reared in simulated sunlight had expression profiles similar to wild-caught individuals, while those reared under standard fluorescent lights (which are substantially red-shifted compared to wild environments) were the same as the lab-bred individuals described by Hofmann et al. [30]. However, the difference in spectral content required to generate plasticity in the lab far exceeded the variation observed in natural light gradients in Lake Malawi (Figure 6). This suggests that although the potential for developmental expression plasticity exists in Malawi cichlids, the expression variation currently observed in shallow, clear-water habitats is probably not the result of environmental influences. Rather, it likely originates in genetic differences in the factors underlying opsin expression. In the event that the developmental environment becomes unstable (as might occur near river mouths during floods or droughts), the environmental change could induce a rapid plastic change in the visual systems of developing fishes.

Opsin expression variation may simply represent genetic “noise” unless there are tangible consequences for neural processing and behavior. A strong link between opsin expression and complex behaviors such as mate choice was not found in bluefin killifish [34]. However, links between performance on an optomotor (OMR) task and both opsin gene sequence [35] and developmental light environment [33, 36] have been demonstrated in cichlid fishes. Smith et al. [33] determined that not only is OMR performance labile but also that this variation in the performance of a luminance-based task are linked to variation in LWS opsin gene expression. This highlights the potential for the generation of behavioral variation via shifts in the expression of cone opsin genes, regardless of whether or not these shifts are environmentally induced. It is important to note, however, that the OMR paradigm is not necessarily an ideal proxy for complex mate-choice behaviors, as it is a luminance-dependent mechanism that does not take into account visual contrast (discussed further in [33]).

## 6. Heterochrony and the Timing of Developmental Variation

In previous work, we have shown that opsin expression can vary through ontogeny and that these shifts appear to be an ancestral trait in African cichlids. By definition, ontogenetic shifts represent changes in developmental programs that are finalized prior to adulthood, that is, although variation will be observed across juveniles of different ages, adult animals with the same developmental program will be relatively homogenous. Data from the tilapia *Oreochromis niloticus* demonstrate that, for this species, the visual system progressively passes through the short-, medium-, and long-wavelength trichromatic opsin gene expression palettes from hatching to adulthood (~6 months; [37]). Heterochronic shifts in developmental timing result in (i) the retention of the neotenic short-wavelength template, (ii) direct expression of the long-wavelength palette, or (iii) accelerated development of the medium-wavelength palette [37]. For the sake of future discussion, we will define any period for opsin expression variation as the critical period and the achievement of the adult phenotype as crystallization per the literature on other phenotypically plastic traits (birdsong; reviewed by [38]).

**Figure 7:**
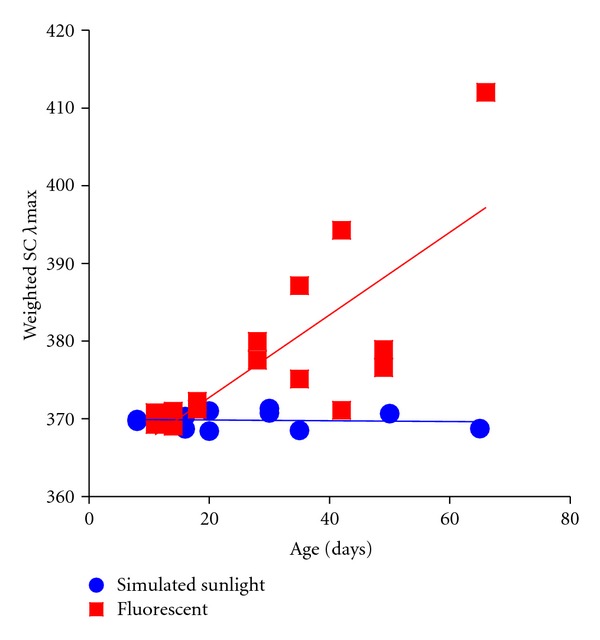
Change in the relative expression of the SWS gene group (SWS1, SWS2B, SWS2A) plotted using a weighted *λ*_max⁡_ calculation through developmental time for *Melanochromis johanni* “black and white.” For this species, an increase in the weighted *λ*_max⁡_ indicates a shift from SWS1 to SWS2B expression. Individuals reared under simulated sunlight exhibit little plasticity through development, while individuals reared under fluorescent lighting express more SWS2B over time. Also, there was much greater quantitative variation in SWS1/SWS2B expression in fish raised under fluorescent lighting.

The variation of gene expression through ontogeny in the lab suggests that the critical period required to achieve adult expression profiles in Malawi cichlids may vary between species. For example, Smith et al. [33] used two species in their study; one species exhibited environmentally induced plasticity (*Metriaclima lombardoi*) while the other did not (*Melanochromis auratus*). In the case of *M. auratus*, the final adult phenotype develops between 11 and 14 days after fertilization (dpf), with a shift from the expression of SWS1 to SWS2B. This switch in SWS gene expression occurred at the same developmental time point in both the broad- and narrow-spectrum laboratory light environments and appears to be ontogenetically fixed. For *M. lombardoi*, changes in gene expression were observed steadily through development over a period of four to six months, at which point the adult phenotype crystallized. The rate at which expression of the LWS pigment increased during the critical period differed between simulated sunlight and fluorescent lighting, resulting in adult phenotypes with differing levels of LWS expression [33]. If we consider developmental progressions in another known plastic species from the laboratory, we see a similar pattern in a different opsin gene. In *Melanochromis johanni* “black and white,” SWS2B expression increases through time until crystallization, and the rate at which it increases determines the adult phenotype (Figure 7). This phenomenon is such that the ontogeny of gene expression in *M. lombardoi* and *M. johanni* “black and white” is similar, although the latter is a congener to a developmentally fixed species (*M. auratus*). This suggests that heterochrony can vary between closely related species and that ontogenetic changes in gene expression can interact with environmental plasticity to produce distinct adult phenotypes.

## 7. Evolution and the Cooption of Heterochronic Variation

With the substantial variation in visual systems and signals present in Lake Malawi, the question of which evolutionary factors are at play remains to be addressed. As previously discussed, the ambient light environment in Lake Malawi probably does not exert significant stabilizing or diversifying selective pressures on the various shallow water visual templates found in Lake Malawi. This would allow the genetic and neural capacity for visual variation to remain in natural populations as a neutral trait under stable environmental conditions. Intuitively, the second ecological factor that could select for specific visual characteristics is the habitat choice and dietary requirements of individual species. An association does exist between diet and SWS1 expression across a panel of distantly related Malawi cichlid species [12]. The resulting UV sensitivity has also been found to be important for foraging [39]. However, we found no link between ecology and expression of the other opsin genes, in particular the RH2 and LWS genes, suggesting that ecology does not explain diversity at the long-wavelength end of the spectrum. Further, functional ecological diversity within genera is fairly limited. Given that this is the phylogenetic level at which most diversification in visual signals has evolved, it is unlikely that ecology is the primary selective force on the visual system as well. 

To the extent that visual systems are largely unconstrained by environmental and ecological selection pressures in Lake Malawi, the potential for “non-adaptive” evolutionary forces increases substantially. In particular, genetic drift could act to generate or limit diversity by randomly altering the nature of ontogenetic variation. Population sizes of Lake Malawi cichlids have been estimated to be as small as 3000 to 5000 individuals suggesting that drift could act with sufficient efficiency to fix nonadaptive alleles [40]. By turning the heterochronic critical period “on” or “off” or simply changing its duration, drift could drastically alter crystallized adult phenotypes without requiring changes to opsin sequences or the basic transcription machinery that governs opsin expression. If we compare this prediction with measurements of gene expression from wild populations, we hypothesize that the qualitative gain or loss of a critical period within a single species could generate two qualitatively different adult expression phenotypes, such as that observed for both *Mchenga eucinostomus* and *Metriaclima zebra* in the wild (see Figure 2 of [14]). Two populations of *M. eucinostomus* were found to vary in LWS expression, while populations of *M. zebra *were found to vary in SWS2b expression, perhaps as a result of changes to genetic factors underlying ontogenetic patterns of gene expression. Similarly, changes in the duration of a critical period could generate extensive quantitative variation based on the total time an individual's visual system can progress through a developmental shift before crystallization. This prediction would match the phenomenon observed in two populations of *Tropheops gracilior* that were sampled along a steep depth gradient (see Figure 2 of [14]) as well as variation in gene expression in *Melanochromis johanni *“black and white” in the laboratory (Figure 7). Here, gene expression is presented by converting it to weighted single cone *λ*_max⁡_, using

(2)λSC=fSWS1λSWS1+fSWS2bλSWS2b+fSWS2aλSWS2afSWS1+fSWS2b+fSWS2a,

where *λ*_*i*_ is the peak sensitivities previously measured (*λ*_SWS1_  = 368 nm, *λ*_SWS2b_  = 423 nm, and *λ*_SWS2a_ = 455 nm [41]) and *f*_*i*_ are the gene expression fraction for the three single cone genes, SWS1, SWS2b, and SWS2a. Both variation in individual gene expression patterns with depth or differential environmental lighting in the lab could generate behavioral shifts such as those observed in the optomotor response, regardless of whether the differences in gene expression occurred in response to environmental conditions or if it was genetically programmed [33]. Perhaps most importantly, critical period changes could generate similar patterns of diversity by a random pattern of changes in the length of the critical period. This would explain the continual reevolution of phenotypic diversity that we observe in African cichlids [37] while accommodating the lack of major selection pressures as posited by Smith et al. [14]. While these developmental changes account for the generation of substantial diversity in sensory systems and nuptial displays, the tendency of the sensory system to evolve free of environmental selection is inconsistent with the fundamental premise of the sensory drive hypothesis. Rather, it is more consistent with the sensory exploitation model of Ryan and Rand [20].

## 8. What Does Sensory Diversity Mean for Signalers?

The third and final tenet of the sensory drive hypothesis centers on the evolution of male courtship displays to match female sensory traits and potential coevolution of these two traits in concert. For example, in Lake Victoria males of different species have evolved either blue or red nuptial coloration based partially on the environment and the visual systems of females [26]. In this system, strong selection would cause females within a species/population to have very similar sensory capacities due to the need to retain sensitivity in a given environment. Therefore, male signals can more easily evolve to match those sensitivities closely. However, in some systems intrapopulation expression variation can be fairly extensive. For example, opsin gene expression in *Tropheops gracilior *collected from 20 m depth in Lake Malawi was significantly more variable than their conspecifics collected at 5 m [14]. In this example, female sensory capacities at 20 m would be a relatively unknown quantity to a courting male. This could create problems for males that attempt to mate with as many females as possible and has implications for signaling systems.

As many recent studies have highlighted, courtship in African cichlids is a multimodal affair. In particular, males often employ acoustic signals as part of their courtship dance [9, 11, 17]. These calls may be used to differentiate species based on call qualities such as frequency and duration [11, 18], but there is often extensive variation in call characteristics within species and even within successive signals from a focal individual [9]. This acoustic plasticity is further compounded by variation in the association of visual and acoustic signals [9], resulting in what van Staaden and Smith [42] posited is a complex communication system that may reflect contextual information, indicate male motivation, or even exploit a female's sensory system.

**Figure 8:**
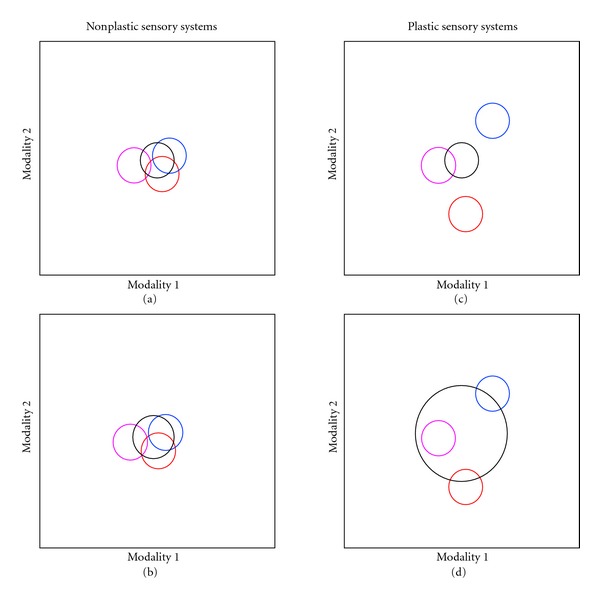
Illustrative cartoon of the effects of sensory plasticity on multimodal male courtship signals. In all panels, the male signal is indicated by the black circle and the female “choice zone” (i.e., the male signal that will elicit a positive response) for three individuals are indicated via colored circles. In the case of a nonplastic species (a), a static male signal will likely overlap the choice criteria for all females, and even a small expansion of male behavior through plasticity will result in effective stimulation of all females involved (b). However, in the case of a plastic species with highly distributed female preferences, a static male signal is unlikely to overlap many female choice zones (c). In order to overlap the choice zones for multiple females, a male must therefore employ a very plastic signal to increase his odds of eliciting a positive response from a given female (d).

In principle, males could use complex communication to account for uncertainty in the preferences of multiple females. If this is indeed the case, we would hypothesize that males of species with variable sensory systems would exhibit more variable multimodal displays (concept illustrated in Figure 8). While no study has directly tested this to date, anecdotal evidence suggests that this is a question worth pursuing. Smith et al. [33] found that *Metriaclima lombardoi* had extensive variation in LWS expression and that this translated into substantial differences in behavioral measures of visual sensitivities. Smith and van Staaden [9] found that males of this species had highly variable acoustic and multimodal courtship displays. Similarly, *Melanochromis auratus* did not have variable opsin expression and little variation in their visual sensitivities on the OMR task [33]. Behavioral trials indicated that, while this species can vocalize, they rarely do so during courtship and therefore have fairly static unimodal displays [9]. Taken together, this suggests that the complexity of male signals in Lake Malawi may be a response to sensory variation. Although intriguing, this idea is admittedly speculative and will need to undergo extensive and rigorous testing.

## 9. Conclusions and Suggestions for Future Research

**Figure 9:**
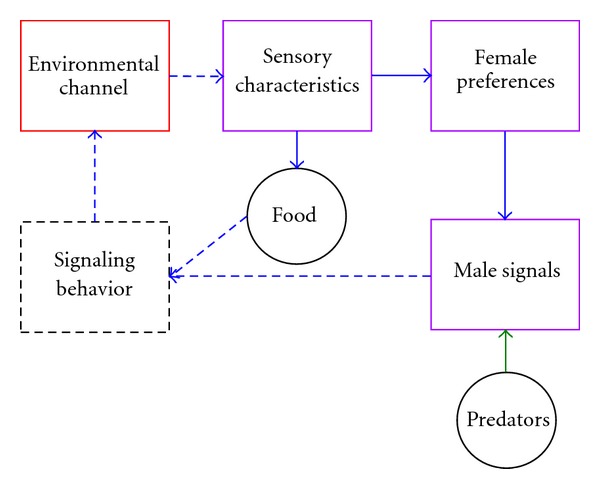
A modified version of the sensory drive schematic proposed by Endler [21]. Violet boxes denote the portions of the model that coincide with the original sensory bias hypothesis first proposed by Ryan and Rand [20]. The red box represents the primary driver of sensory stabilization or diversification (the environmental transmission channel). Solid arrows represent selective forces that are likely active in Lake Malawi, while dashed arrows represent factors whose selective influence on sensory evolution in Lake Malawi is marginalized due to environmental characteristics.

**Figure 10:**
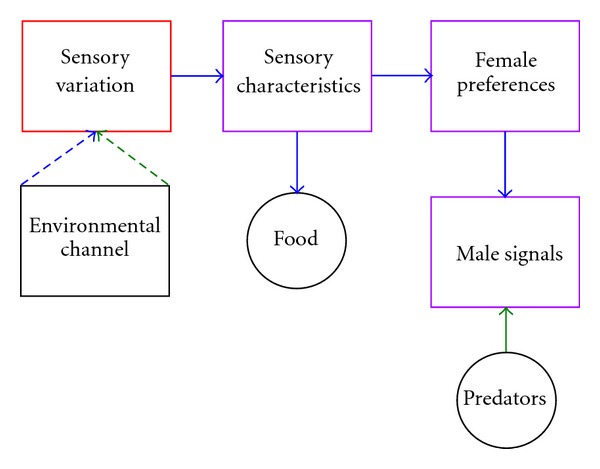
A further modification of the sensory drive framework that inserts sensory plasticity as a buffer between the environmental transmission channel and the sensory bias framework. Sensory plasticity is highlighted in red due to the potential for modulating variation in sensory sensitivities either independent of or in response to environmental effects.

In order to summarize the ideas presented here, it is useful to revisit the original scheme for sensory drive so effectively laid out by Endler [21], layering in how facets of Lake Malawi cichlid biology relate to specific portions of his model (Figure 9). The sensory drive model is dominated by the qualities of the environment, and how these qualities influence sensory systems and communication. In the absence of strong environmental influences, many of the aspects of the model become less influential (as depicted by dashed arrows). If we remove these portions from the model, we can begin to see in which way the Lake Malawi system is unique (Figure 10). So where, in fact, does sensory variation fit in this model? In essence, data suggest that sensory variation, which can function in conjunction with or independent of the environment depending on the species and habitat, acts as a buffer between the “environmental channel” and “sensory characteristics” portion of the sensory drive model.

As an example, let us again consider the *Mchenga eucinostomus *collected at two different depths along a habitat gradient in Lake Malawi. These fish exhibited profound visual plasticity, with fish from 5 m depth being tetrachromats (SWS1-RH2B-RH2A-LWS) while fish from 20 m were trichromats (SWS1-RH2B-RH2A). Quantum catch models predict that this qualitative variation in LWS opsin expression would have no effect on luminance function for shallow littoral Lake Malawi habitats (i.e., the retina will not catch more light), although enhancing luminance detection is likely a driving selective force in Lake Victoria [14]. However, an increase in LWS function in Malawian taxa is known to have behavioral consequences in the OMR paradigm, with an increase in LWS expression increasing behavioral response/sensitivity [33]. This corresponds to a change in function of a particular neural pathway: the magnocellular visual pathway. Therefore, we can deduce that sensory variation in Lake Malawi cichlids is capable of generating variation in the behavior of wild fish such as that observed in Victorian cichlids [35]. However, this variation in Malawi can occur without the need for the same selective gradients that would be present for the same fish in Lake Victoria.

Although sensory variation in contemporary Malawi cichlid biology is clearly discernible, many avenues of research remain to determine exactly how important variation has been in cichlid evolution through time. Aside from further profiles of sensory variation in Lake Malawi, we envision two particularly important avenues of future research: (i) investigations of the role of variation in the cichlid tribes of Lake Tanganyika and (ii) studies of the molecular and cellular mechanisms involved in the stabilization of the developing retina. The former represents an important opportunity to test whether variation also functions in independent cichlid radiations. Since Tanganyikan cichlids represent multiple independent lineages that have evolved in a visual environment more similar to Lake Malawi than Lake Victoria, they may elucidate mechanisms of sensory evolution in relatively nonrestrictive environments. Indeed, work by Sugawara et al. [43] on the cichlid rod opsin suggests parallel evolutionary processes in Lakes Malawi and Tanganyika with respect to rod cellular sensitivities. The molecular and cellular mechanisms important in the developing retina are of considerable importance to the broader field of neuroscience, as mechanisms determining vertebrate neural plasticity are of great significance for both basic and clinical research.

In sum, we propose that sensory variation is quite diverse in the Lake Malawi cichlids. However, we cannot explain this diversity by simple models of sensory drive. Such variation could dramatically alter the nature and pace of sensory evolution and visual communication, though correlations between cichlid color and sensory variation have yet to be demonstrated in Lake Malawi. Given the potential for sensory variation to modify our understanding of both sensory evolution and cichlid speciation, contemporary research should consider the implications of these mechanisms when interpreting experimental results. Not only would a fresh view of cichlid communication biology further emphasize the importance of these fishes as evolutionary models, it could also be an important model for questions in the neurosciences.
